# The biomechanical effect of tibiofemoral conformity design for patient-specific cruciate retainging total knee arthroplasty using computational simulation

**DOI:** 10.1186/s40634-019-0192-6

**Published:** 2019-06-03

**Authors:** Yong-Gon Koh, Kyoung-Mi Park, Kyoung-Tak Kang

**Affiliations:** 1 Joint Reconstruction Center, Department of Orthopaedic Surgery, Yonsei Sarang Hospital, 10 Hyoryeong-ro, Seocho-gu, Seoul, 06698 Republic of Korea; 2 Department of Mechanical Engineering, Yonsei University, 50 Yonsei-ro, Seodaemun-gu, Seoul, 03722 Republic of Korea

**Keywords:** Total knee arthroplasty, Patient-specific implant, Conformity

## Abstract

**Background:**

Alterations to normal knee kinematics performed during conventional total knee arthroplasty (TKA) focus on the nonanatomic articular surface. Patient-specific TKA was introduced to provide better normal knee kinematics than conventional TKA. However, no study on tibiofemoral conformity has been performed after patient-specific TKA. The purpose of this study was to compare the biomechanical effect of cruciate-retaining (CR) implants after patient-specific TKA and conventional TKA under gait and deep-knee-bend conditions.

**Methods:**

The examples of patient-specific TKA were categorized into conforming patient-specific TKA, medial pivot patient-specific TKA and anatomy mimetic articular surface patient-specific TKA. We investigated kinematics and quadriceps force of three patient-specific TKA and conventional TKA using validated computational model. The femoral component designs in patient specific TKA were all identical.

**Results:**

The anatomy mimetic articular surface patient-specific TKA provided knee kinematics that was closer to normal than the others under the gait and deep-knee-bend conditions. However, the other two patient-specific TKA designs could not preserve the normal knee kinematics. In addition, the closest normal quadriceps force was found for the anatomic articular surface patient-specific TKA.

**Conclusions:**

Our results showed that the anatomy mimetic articular surface patient-specific TKA provided close-to-normal knee mechanics. Other clinical and biomechanical studies are required to determine whether anatomy mimetic articular surface patient-specific TKA restores more normal knee mechanics and provides improved patient satisfaction.

## Background

The treatment of severe knee joint arthritis with total knee arthroplasty (TKA) continues to grow and a wide range of implants based on various designs are available on the market (Greene 2007; Losina et al. 2012; Stoddard et al. 2013). Although the survival rate of TKA has been excellent, the reported patient satisfaction has not been as good as that of other orthopaedic treatments such as total hip arthroplasty, with more than 20% of TKA patients reporting dissatisfaction with regard to implant outcomes (Anderson et al. 1996; Baker et al. 2007; Mannion et al. 2009; Zeller et al. 2017). This dissatisfaction is caused by anterior knee pain, mid-flexion instability, a reduction in the range of flexion and the incomplete return of function (Devers et al. 2011; Noble et al. 2005; Parsley et al. 2010).

TKA that returns proper functions tends to assuage the most common criticisms regarding pain and stiffness, leading to the belief that TKA should restore “normal-like” kinematic functions (Zeller et al. 2017). Therefore, there has been interest in the development of innovative TKA implants, with which advanced surgical technologies could provide more normal-like functions for the knee joint, for an increasingly younger and more active patient population (Jaffry et al. 2014; Walker et al. 2014).

The recent technologies of magnetic resonance imaging (MRI) and computer tomography (CT) scans are being used to provide data for manufacturing a patient-specific TKA or instrumentation. There are advantages to both methods, but each is better suited to a particular type of joint analysis (Slamin and Parsley 2012). The goal of patient-specific TKA is to optimize the bony coverage and provide articulating surfaces close to the subject’s natural anatomy, corrected for any underlying deformity (Slamin and Parsley 2012). However, current patient-specific related studies have only customized the femoral component of a patient’s anatomy, followed by conforming the design of the tibial insert to that femoral component (ConforMIS). These patient-specific TKA procedures have shown more normal femoral rollback in in-vitro study, but there has been no study that investigated the effect of the tibial articular surface in relation to the post-operative kinematics (Patil et al. 2015).

The purpose of this study was to investigate the effect of the articular surface conformity on patient-specific posterior cruciate-retaining (CR) TKA, along with conventional CR TKA. The patient-specific TKA procedures were categorized into conforming patient-specific TKA, medial pivot patient-specific TKA and anatomy mimetic articular surface patient-specific TKA. We investigated four different types of TKA to compare the kinematics and quadriceps force with a normal model under gait and deep-knee-bend conditions using finite element (FE) simulation. We hypothesized that anatomic articular surface TKA provides the most normal-like mechanics.

## Methods

### Design of patient-specific CR TKA

**Fig. 1:**
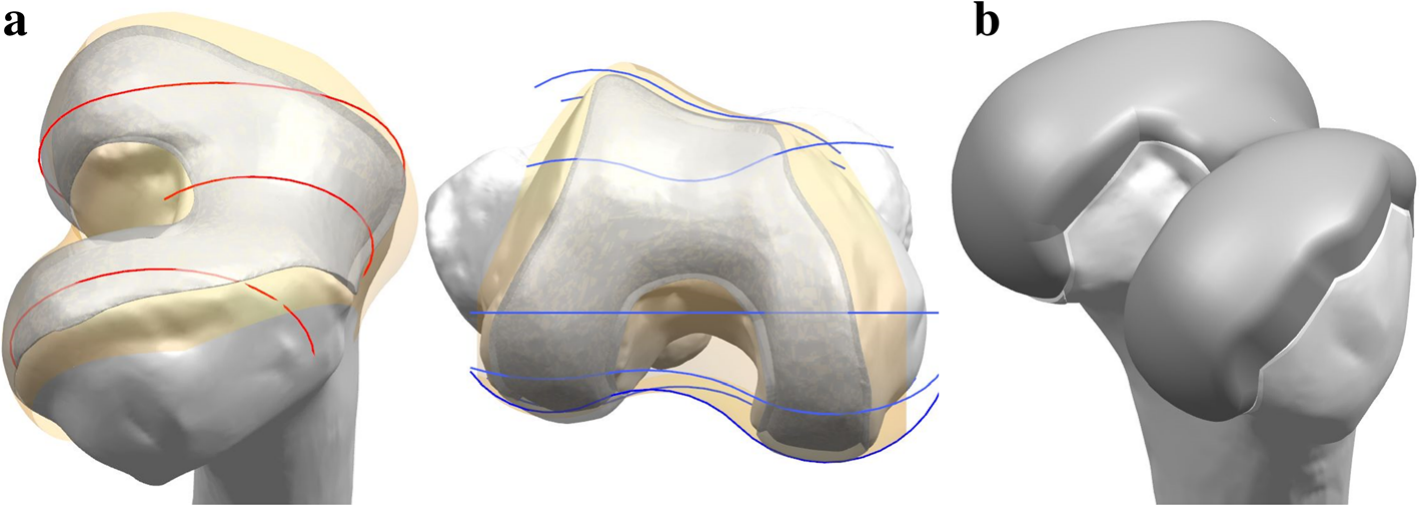
The method of development for patient-specific femoral component: **a** patient-specific curves from sagittal and coronal planes used in the design; **b** developed patient-specific femoral component

Patient-specific TKA was designed with a previously existing three-dimensional (3D) knee joint model (Kang et al. 2017c, 2018). The patient-specific design was initiated by the acquisition of CT and MRI scans of a patient’s knee joint. The image data were imported into Mimics version 14.1 (Materialise, Leuven, Belgium) for editing and 3D reconstruction. Planes were introduced by the intersection of condyles in both the sagittal and coronal views. Intersection curves were used to extract the articulating surface geometry in both planes, which were imported into Unigraphics NX (Version 7.0; Siemens PLM Software, Torrance, CA, USA) and fitted with rational B-splines (Fig. 1). The three patient-specific J curves for the trochlear grooves and the medial and lateral condyles from the patients’ normal articular anatomy were developed in the Unigraphics NX software (Fig. 1) (ConforMIS; Harrysson et al. 2007; Kurtz et al. 2016; Steklov et al. 2010; Van Den Heever et al. 2011).

A patient’s femur in the coronal plane displays an asymmetry between the lateral and medial condyles. The patient-specific femoral component respects these patient specific differences and is designed with the patient’s natural coronal offset. The coronal offset is defined as the height difference between the medial and lateral femoral condyles, as viewed in the coronal extension plane. This typically creates an asymmetry of the extension gap that must be accounted for at the tibial articular surface. Typically, the lateral posterior condyle is shorter than the medial condyle, which also creates a unique asymmetry in the flexion space. These are the patient-specific design elements, along with the patient’s unique “J” curvatures, that are incorporated into the femoral component (Fig. 1).

The articular geometry of a general patient-specific tibial insert is derived from the femoral component (ConforMIS; Kurtz et al. 2016). The coronal geometry uses a broad radius on both condyles, thus employing the round-on-round principle, which has been shown to reduce contact stress. The coronal conformity is extremely high, yet yields a relatively low constraint design (ConforMIS; Kurtz et al. 2016).

Three different methods were applied to design the articular surface of the patient-specific tibial insert in this study. We applied the tibiofemoral conformity of conventional CR TKA to the patient-specific TKA conformity. To archive this, conforming design TKA systems such as the Genesis II Total Knee System (Smith & Nephew Inc., Memphis, TN, USA) and medial pivot design Evolution Medial Pivot Total Knee Arthroplasty (Wright Medical Technology, Arlington, TN, USA) were selected.

To investigate the CR TKA conformity, they were scanned using a non-contact 3D laser scanner (COMET VZ; Steinbichler Optotechnik GmbH, Neubeuern, Germany) with an accuracy of 50 μm. Scanned point data were converted to 3D models, and scanning was repeated until the 3D model dimensions had geometrical errors of < 100 μm (Kwon et al. 2014).

**Fig. 2:**
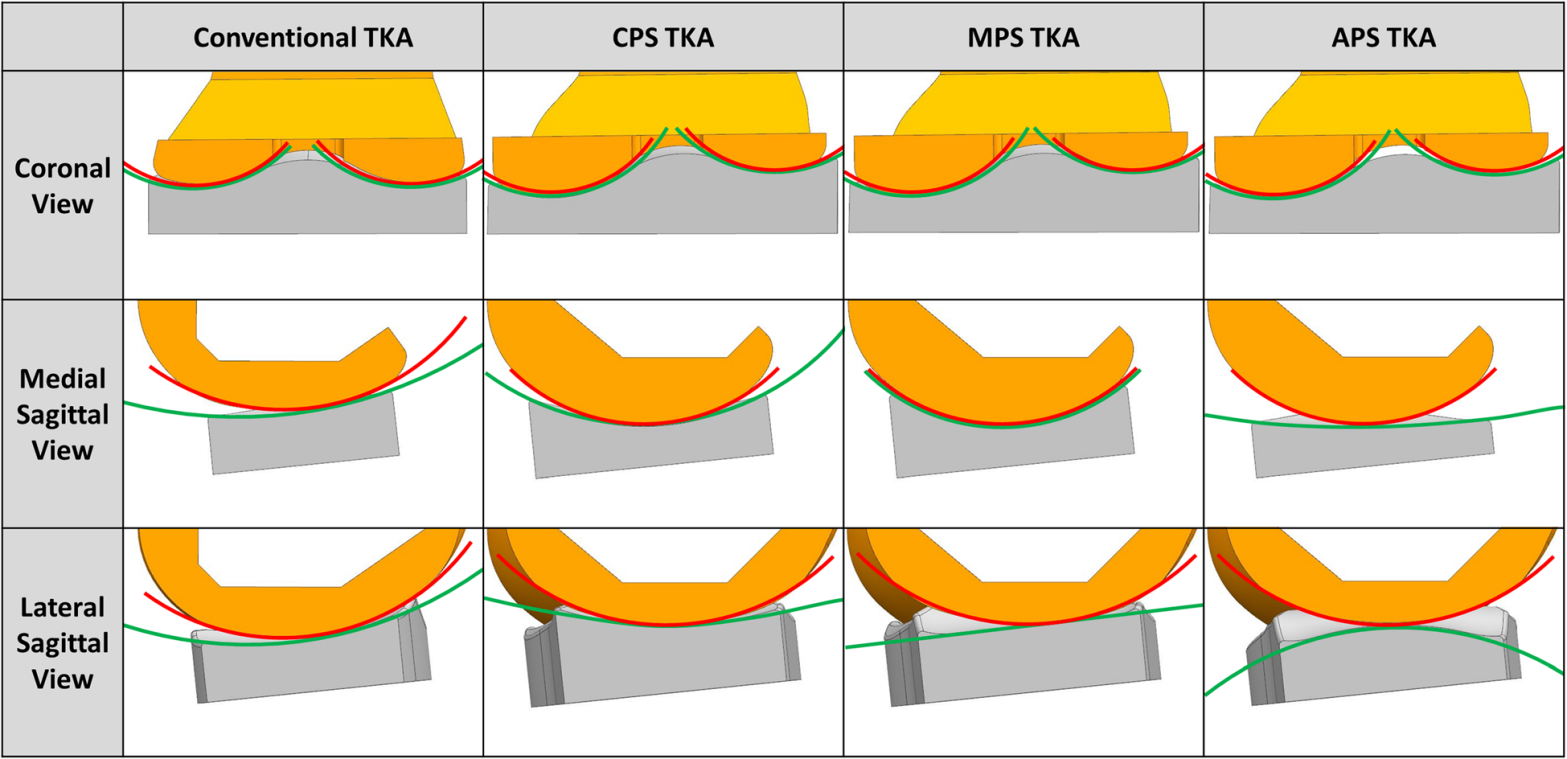
Cross sections of conventional TKA and patient-specific TKAs with three different articular surface conformity

The ratio of the curvature radius of the tibial insert to the curvature radius of the femoral component was investigated to determine the conformity in the coronal and sagittal planes. A tibial insert with conventional CR TKA conformity (Genesis II) and medial pivot tibial insert with medial pivot conformity (Evolution) were developed by applying the curvature radius ratio in the coronal and sagittal planes to the patient-specific femoral component (Fig. 2). In addition, anatomy mimetic patient specific TKA was developed, in which both the femoral and tibial articular surface followed the patient’s geometry. There are three different patient-specific TKA designs, which were categorized as conforming patient-specific TKA (CPS TKA), medial pivot patient-specific TKA (MPS TKA) and anatomic articular surface patient-specific TKA (APS TKA). The femoral component designs in patient specific TKA were all identical.

### Development of normal knee FE model

**Fig. 3:**
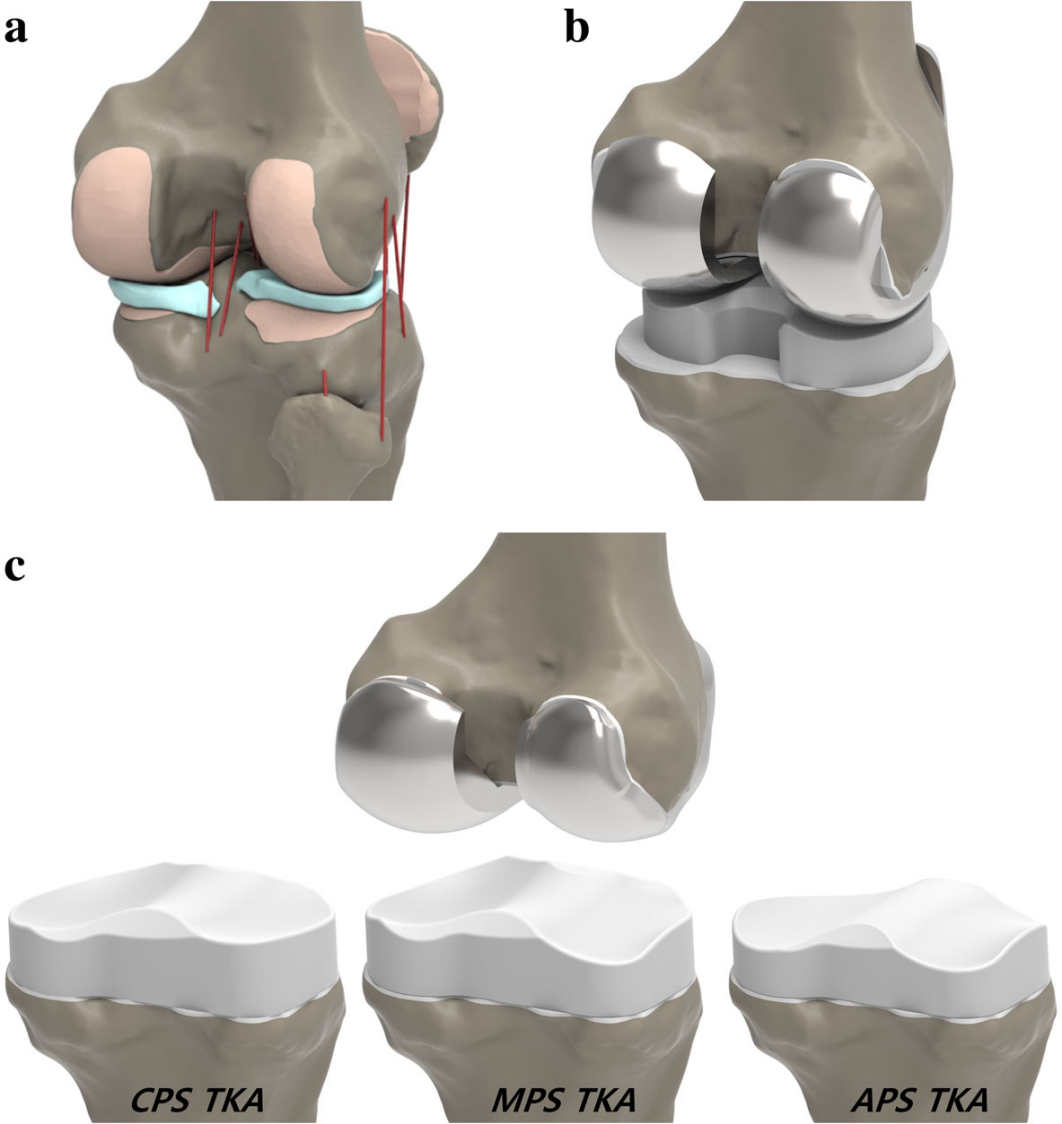
FE model for **a** normal knee, **b** conventional TKA and **c** three different conformity of patient-specific TKA used in this study

As previously mentioned, the existing validated normal knee and TKA model was used in this study (Kang et al. 2016a, 2017a, c, 2018). A 3D non-linear FE model of a normal knee joint was developed using data from CT and MRI scans of a healthy 37-year-old male subject. The model included the femur, tibial, fibular bones, cartilage layers, ligaments and meniscus (Fig. 3).

The bony structures were modeled as rigid bodies (Peña et al. 2006). The articular cartilage and meniscus were modeled as isotropic and transversely isotropic, respectively, with linear elastic material properties (Haut Donahue et al. 2003). In addition, the major ligaments were modeled with nonlinear and tension-only spring elements (Blankevoort and Huiskes 1996; Takeda et al. 1994). The force-displacement relationship based on the functional bundles in the actual ligament anatomy can be represented as follows:

$$ {\displaystyle \begin{array}{c}f\left(\varepsilon \right)=\left\{\begin{array}{c}\frac{k{\varepsilon}^2}{4{\varepsilon}_1},\kern5.5em 0\le \varepsilon \le 2{\varepsilon}_1\\ {}k\left(\varepsilon -{\varepsilon}_1\right),\kern5.25em \varepsilon >2{\varepsilon}_1\\ {}\kern1.5em 0,\kern7em \varepsilon <0\kern1.5em \end{array}\right.\\ {}\varepsilon =\frac{l-{l}_0}{l_0}\\ {}{l}_0=\frac{l_r}{\varepsilon_r+1}\end{array}} $$

where *f*(*ε*) is the current force, k is the stiffness, *ε* is the strain, and *ε*_1_ is assumed to be constant at 0.03. The ligament bundle slack length *l*_0_ can be calculated using the reference bundle length *l*_*r*_ and reference strain *ε*_*r*_ in the upright reference position.

The interfaces between the articular cartilage and bones were assumed to be fully bonded. Six pairs of tibiofemoral contacts between the femoral cartilage and meniscus, the meniscus and tibial cartilage, and the femoral cartilage and tibial cartilage were modeled on both the medial and lateral sides (Kang et al. 2017a). A finite sliding frictionless hard-contact algorithm with no penetration was applied to all the contacts in all articulations (Kang et al. 2017a).

### Development of conventional TKA and patient-specific TKA FE model

Genesis II was used for the conventional TKA FE model. Computer-assisted design models of a CR design from the Genesis II Total Knee System (Smith & Nephew Inc.) were virtually implanted in the bone geometry. Based on the dimensions of the femur and tibia, devices with sizes of 7 and 5–6 were selected for the femoral component and tibial insert, respectively. Conventional and patient-specific TKA models were implanted as shown below. In the neutral position, the femoral component was aligned so that the distal bone resection was perpendicular to the mechanical axis of the femur, and the anterior and posterior resections were parallel to the clinical epicondylar axis in the transverse plane. The tibial default alignment was rotated by 0° relative to the anterior-posterior axis, and the coronal alignment corresponded to 90° relative to the mechanical axis. Similarly, three different patient-specific TKA devices were virtually implanted in the bone geometry (Fig. 3).

Contact conditions were applied between the femoral component, tibial insert and patellar button during TKA. The coefficient of friction between the polyethylene (PE) and metal materials was assumed to be 0.04, for consistency with previous explicit FE models (Wünschel et al. 2011). The materials for the femoral component, PE insert and tibial baseplate were described in previous studies (Godest et al. 2002; Kang et al. 2018).

### Boundary and loading conditions

The loading conditions corresponded to gait and deep-knee-bend loading, which were applied to evaluate the effects of conformity in patient-specific TKA on the generation of normal knee mechanics (Halloran et al. 2010; Kang et al. 2016b; Kutzner et al. 2010; Patil et al. 2015). A computational analysis was performed with an anterior-posterior (AP) force applied to the femur with respect to the compressive load applied to the hip (Halloran et al. 2010; Kang et al. 2017b, 2016b; Kutzner et al. 2010). A proportional-integral-derivative (PID) controller was incorporated into the computational model to control the quadriceps in a manner similar to that in a previous experiment (Kang et al. 2017b). A control system was used to calculate the instantaneous quadriceps displacement required to match a target flexion profile, which was the same as that in the experiment. Internal-external and varus-valgus torques were applied to the tibia (Halloran et al. 2010; Kang et al. 2016b; Kutzner et al. 2010).

The FE model was analyzed using the ABAQUS software (version 6.11; Simulia, Providence, RI, USA). We investigated the kinematics and quadriceps force to evaluate how effectively the generation of normal knee mechanics was achieved for different conformity after patient specific TKA compared to conventional TKA. A three-cylindrical knee joint model was developed with six degrees-of-freedom for the relative kinematics of the tibiofemoral and patellofemoral articulations (Grood and Suntay 1983). Embedded coordinate frames in the femur, tibia and patella were considered using nodes, and their positions were evaluated throughout the loading conditions.

## Results

### Comparison of kinematics in patient-specific TKA and conventional TKA with those in normal knee using FE models

**Fig. 4:**
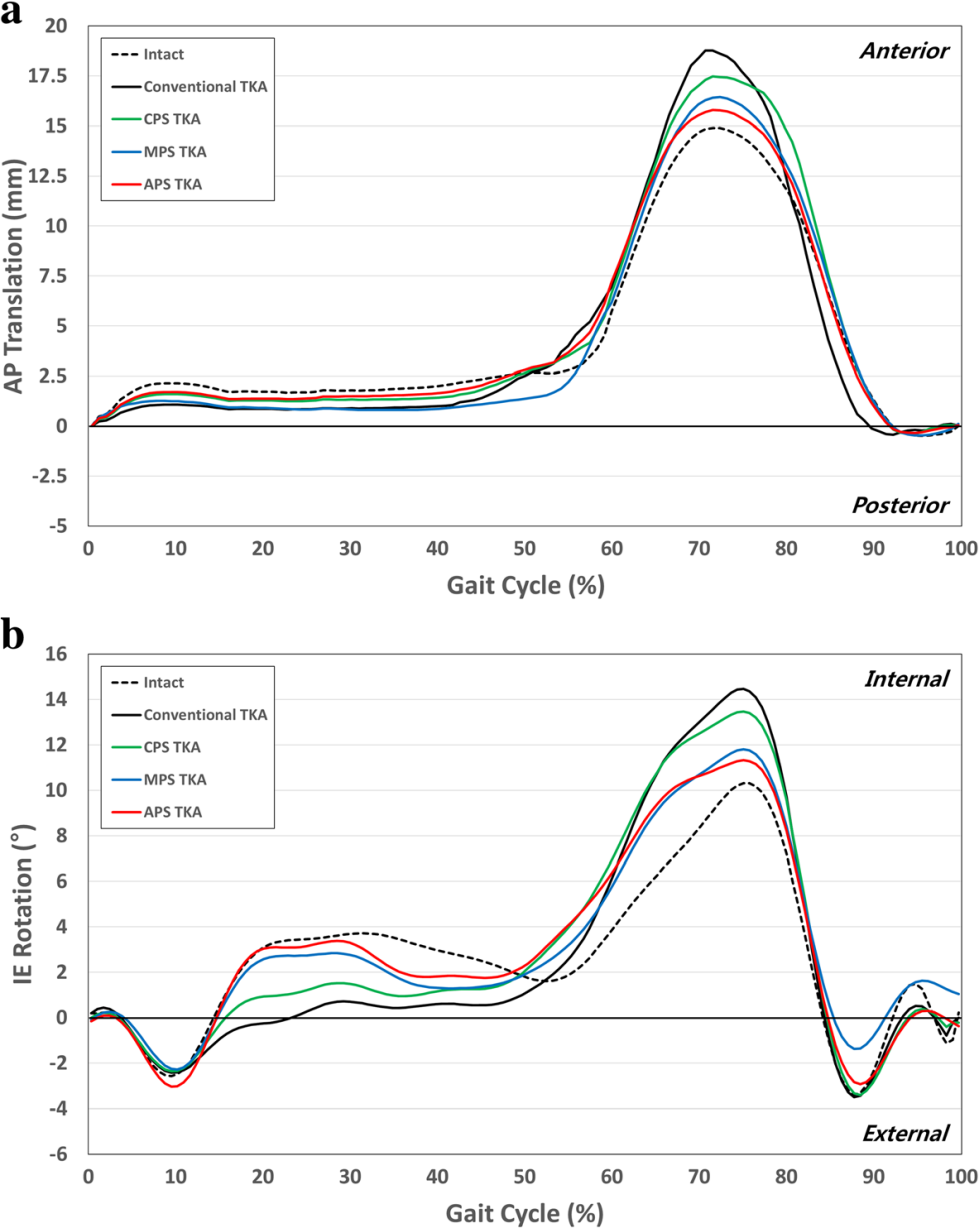
Comparison of **a** AP and **b** IE kinematics between conventional TKA and three different conformity of patient-specific TKA during gait cycle

Figure 4 shows four different TKA and normal knee models with AP and internal-external (IE) rotations under the gait cycle condition. In terms of the AP translation, the four different TKA showed less anterior movement than the normal knee. In particular, this difference was found during the swing phase under the gait cycle condition. Conventional TKA, CPS TKA, MPS TKA and APS TKA showed 3.8 mm, 2.8 mm, 2.1 mm and 1.6 mm greater anterior translation during the swing phase, respectively, compared to the normal knee model. For the IE rotation, the four TKA procedures showed smaller and larger internal rotations during the stance and swing phases, respectively, under the gait cycle condition. Conventional TKA, CPS TKA, MPS TKA and APS TKA showed 4.3°, 3.3°, 2.3° and 1.5° greater internal rotations during the swing phase, respectively, compared to the normal knee model. The kinematics of the APS TKA design was visually closer to the normal AP and IE motions compared to the TKA devices implanted using the other designs.

**Fig. 5:**
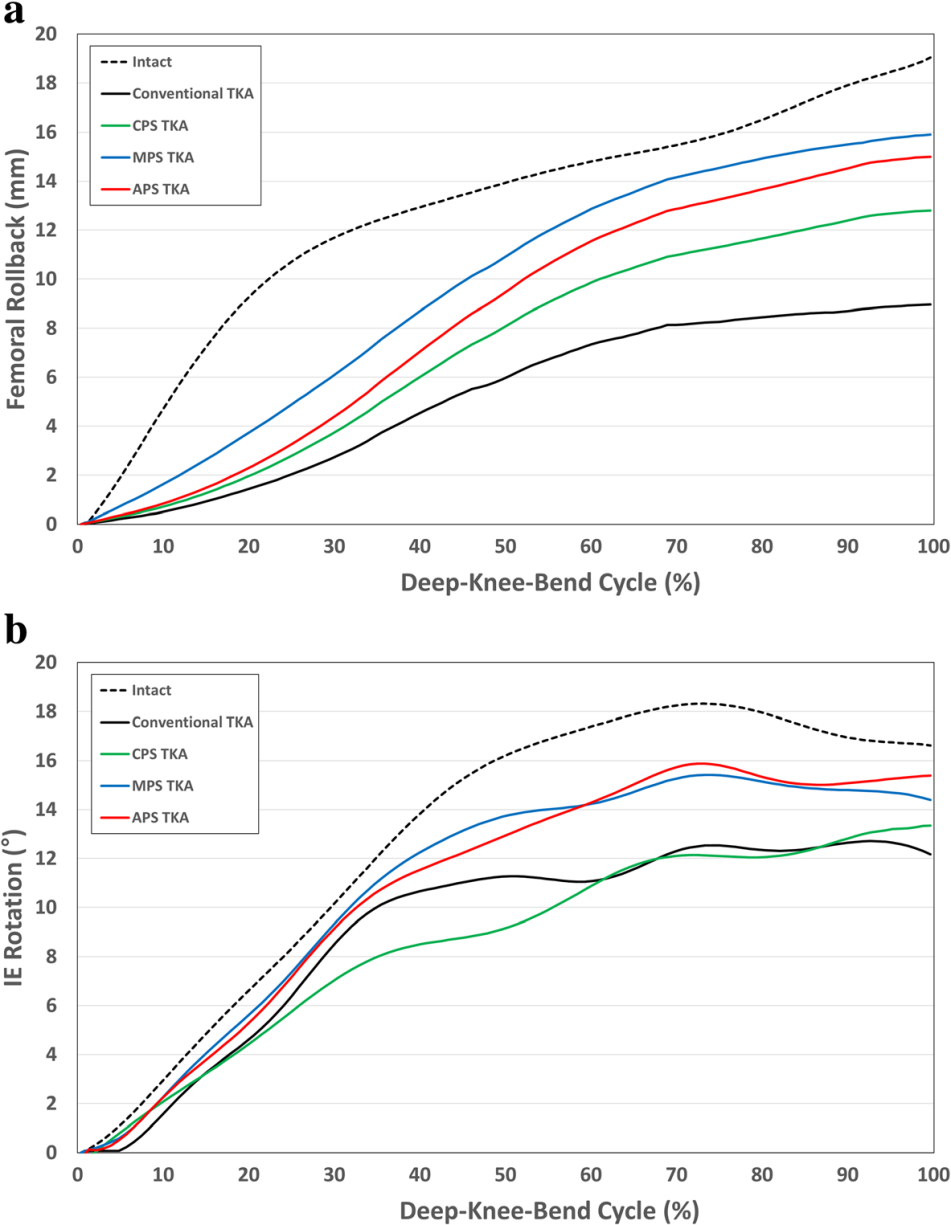
Comparison of **a** femoral rollback and **b** IE kinematics between conventional TKA and three different conformity of patient-specific TKA during deep-knee-bend cycle

Figure 5 shows the values for the rollback and IE rotation in the four different TKA and normal knee models under deep-knee-bend conditions. Similar to the APS TKA under the gait cycle condition, it is visually closer to the normal AP and IE motions compared to the TKA devices implanted with other designs under the deep-knee-bend conditions. There were differences of 9.8 mm and 4.1 mm in the femoral rollback with the conventional TKA and APS TKA, respectively, compared to the normal knee. In addition, the internal rotations were 5.8° and 2.3° smaller with the conventional TKA and APS TKA, respectively, compared to the normal knee under the deep-knee-bend conditions.

### Comparison of quadriceps force in patient-specific TKA and conventional TKA with those in normal knee using FE models

**Fig. 6:**
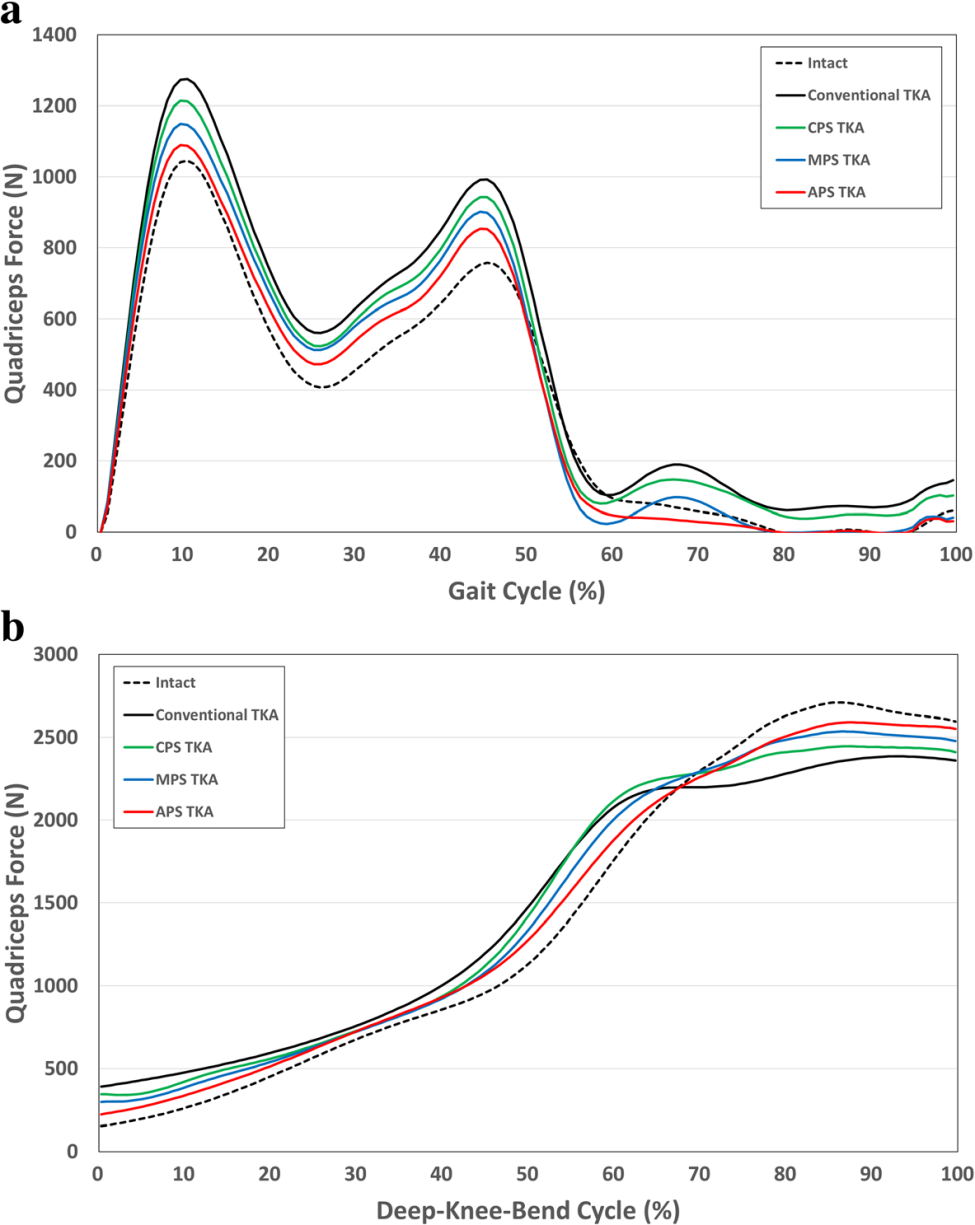
Comparison of quadriceps force between conventional TKA and three different conformity of patient-specific TKA (CPS TKA, MPS TKA, APS TKA) during **a **gait and **b** deep-knee-bend cycle

Figure 6 showed the quadriceps force values in the four different TKA and normal knee models under the gait and deep-knee-bend conditions. All four TKA models showed greater quadriceps force values than the normal knee during the gait cycle. Such a trend was also found under the deep-knee-bend conditions, particularly at less than 70°. However, the quadriceps force was smaller than the normal knee under high flexion (> 80°) in all four different TKA models. In addition, APS TKA showed the least difference in the quadriceps force compared to the other TKA designs.

## Discussion

The most important finding of this study was that the anatomy mimetic tibial articular surface APS TKA showed knee mechanics that were the closest to normal knee joint. Other patient-specific TKA designs also showed knee kinematics that was closer to normal than that with conventional TKA, but their tibial inserts were derived from the femoral component. Thus, they were not as effective as APS TKA in terms of normal knee kinematics.

Patil et al. showed that patient-specific implants generated kinematics that more closely resembled normal knee kinematics than standard knee designs using an in-vitro cadaveric experiment (Patil et al. 2015). In addition, Koh et al. used computational simulation to show that the restoration of the normal geometry of the knee joint in patient-specific bicruciate-retaining TKA and the preservation of the anterior cruciate ligament led to an improvement in the kinematics compared with the conventional CR and bicruciate-retaining TKA (Ivie et a. 2014). Moreover, recent clinical studies showed comparative results between patient-specific TKA and conventional TKA (Ivie et al. 2014; Wang et al. 2017; Zeller et al. 2017). Ivie et al. reported that the reliable reproduction of a limb’s mechanical axis may accrue from patient-specific TKA compared to standard, intramedullary instrumentation (Ivie et al. 2014). Wang et al. suggested that patient-specific bi-compartmental arthroplasty is a viable treatment option and may contribute to superior mechanical advantages (Wang et al. 2017). Furthermore, Zeller et al. recently demonstrated that the kinematics after patient specific TKA was similar to that of a normal knee (Zeller et al. 2017). Therefore, using customized implant technology through patient-specific TKA designs affords benefits, including more normal motion compared with conventional TKA (Zeller et al. 2017). However, all of the previously mentioned studies investigated a tibial insert design derived from the femoral component in patient-specific TKA. (Koh et al. 2017; Wang et al. 2017; Zeller et al. 2017) A previous study showed that the articular geometry of a knee implant has a competing impact on the kinematics and contact mechanics of conventional TKA such that a geometry with lower contact pressure will impose more constraints on the knee kinematics (Ardestani et al. 2015). In other words, changes in the conformity of the femoral and tibial insert can impact the performance metrics (Ardestani et al. 2015).

The objective in our study was to determine the effect of femoral and tibial conformity in patient-specific TKA using computational simulation. The advantage of computational simulation using a single subject, as used in this study, was the ability to determine the effects of conformity on the tibiofemoral articular surface for an identical subject, without the effect of variables such as the weight, height, bony geometry, ligament properties and component size (Thompson et al. 2011). The intact knee model was validated, and the results showed good agreement with experimental data for the kinematics and contact area, as demonstrated by the FE analysis of the identical subject (Kang et al. 2016a, 2017a). In addition, conventional TKA models have been validated using kinematic data from previous experimental results (Wünschel et al. 2011). Therefore, the patient-specific TKA model developed in this study, along with the following analysis, can be considered reasonable.

We found that patient-specific TKA showed knee kinematics that was closer to normal than conventional TKA under gait and deep-knee-bend conditions (Thompson et al. 2011). In addition, patient-specific TKA with anatomy mimetic articular surface conformity showed the most normal-like kinematics. These findings are consistent with those of a previous study using computational simulation with mimetic articular surface design TKA (Varadarajan et al. 2015). Such APS TKA surfaces are compatible with the normal knee kinematics and soft tissue envelope. Geometric comparisons of the APS TKA surface to contemporary designs showed that the articular surfaces of TKA were fundamentally incompatible with normal knee motion. Further, kinematic simulations showed that the anatomic geometry of the APS TKA surface directly contributed to the restoration of normal knee kinematics. In addition, a previous study noted paradoxical anterior sliding from 0° to 45° of flexion, reduced femoral rollback, and a significant reduction in the tibial internal rotation in patients with conventional TKA compared to normal subjects (Yue et al. 2011).

An interesting finding was that it showed knee kinematics that was closer to normal compared to MPS TKA and CPS TKA. The traditional “medial pivot” implant on the medial side is designed to be a “ball-in-socket” articulation (Blaha 2004). Previous studies showed that a normal knee has a minimum movement of the medial femoral condyle and a posterior translation of the lateral femoral condyle during flexion; this movement was called “medial pivot” (Asano et al. 2001; Dennis et al. 2005; Hill et al. 2000). Therefore, the above result could be found. However, APS TKA with the perfect articular surface of a normal knee could not restore the normal kinematics because of the absence of the anterior cruciate ligament (ACL). In a natural knee joint, the ACL and asymmetric shape of the tibial articular surface contribute to the controlled differential medial/lateral femoral rollback, AP translation, IE rotation and activity dependent kinematics (Zumbrunn et al. 2015). The abnormal posterior femoral location in CR TKA is largely due to the absence of the ACL, which is under tension in extension and holds the femur anteriorly on the tibia. Following this posterior shift, the force imbalance within the joint causes paradoxical anterior sliding of the femur during early flexion (Zumbrunn et al. 2018). In addition, a greater internal rotation was found compared to a normal knee during the swing phase because it experienced non-weight bearing motion, unlike the stance phase in the gait and deep-knee-bend motion.

Theoretically, a TKA design with the center of the flexion located relatively more posterior could result in additional lengthening of the quadriceps lever arm. A previous study showed that a longer lever arm reduces the tension on the quadriceps during knee extension, especially at flexion angles that typically generate high knee moments (D’Lima et al. 2001). Because osteoarthritis (OA) TKA patients experience significant quadriceps weakness, increases in the required quadriceps force could make it more difficult for patients to kneel, squat or rise from a chair (Mizner et al. 2005; Thompson et al. 2011). Our result showed that after TKA, the knee required more quadriceps force under the gait cycle. These results contradicted those of Li et al. (2013), who observed post-operative gait adaptations such as “quadriceps avoidance” in TKA patients (Li et al. 2013). However, they stated that the TKA patients compensated for this deficiency by leaning their trunks forward. Our FE model showed different results for the quadriceps force in the PID controlled flexion, and the trunk could not guarantee the same flexion. In addition, more and less quadriceps force were required in the TKA model for the low and high flexion, respectively, similar to a cadaveric experiment under deep-knee-bending (D’Lima et al. 2001). A previous study showed that the quadriceps ratios from the isokinetic testing of these three prosthesis design groups were greater than those of the healthy group (Li et al. 2013). They showed that the quadriceps ratios after successful TKA were not the same as those of the healthy group even after a long period (6–13 years) of functional adaptation (Li et al. 2013).

In terms of biomechanical aspect, the APS TKA design showed the nearest normal knee biomechanics during gait and squat conditions. However, the APS TKA design did not fully implement normal knee biomechanics. The main reason could be the absence of anterior cruciate ligament. In addition, the development of materials of implants that implement real biomechanics of anatomical knee joints will be needed.

There were several limitations to the current study. First, gait and deep-knee-bend simulations were performed, although simulations related to more demanding activities such as chair rising, sitting, stair climbing and stair descending are required for a more reliable investigation. Second, the implant kinematics and quadriceps force were evaluated using computational simulations, which did not fully represent in-vivo conditions. Third, the computational model was developed using data from a single subject because the time and computational cost associated with generating many subject-specific FE models made doing so inefficient. Additionally, although the FE model was well validated, it did not represent an in-vivo environment by considering anatomic variations and age-related changes in the ligament and cartilage. Thus, it is necessary to expand the number of subjects in future research. This approach is widely used in orthopedic biomechanics (Kang et al. 2017b, c, 2018; Koh et al. 2017; Kwon et al. 2014; Thompson et al. 2011; Varadarajan et al. 2015; Zumbrunn et al. 2018; Zumbrunn et al. 2015). Moreover, we validated the intact and conventional TKA models using the results of a previous experiment. Finally, the results were unable to substitute for clinical results and consider patient satisfaction because they corresponded to the outcomes of computational analyses. However, the main factor analyzed in the present study corresponded to the main components being investigated to evaluate a biomechanical effect in computational biomechanics (Kang et al. 2017b, c, 2018; Koh et al. 2017; Kwon et al. 2014; Thompson et al. 2011; Varadarajan et al. 2015; Zumbrunn et al. 2018, 2015).

## Conclusions

 We evaluated conventional TKA and patient-specific TKA with respect to different conformity levels and determined the design that provided the most normal mechanics. We found that APS TKA with an anatomic articular geometry was able to more closely mimic the activity dependent kinematics of normal knees. Moreover, the quadriceps force was also most similar to that of a normal knee after APS TKA. Other clinical and biomechanical studies are required to determine whether anatomic articular surface patient-specific TKA restores more normal knee mechanics and improves patient satisfaction.

## Abbreviations

3D: Three-dimensional
ACL: Anterior cruciate ligament
AP: Anterior-posterior
APS TKA: Anatomic articular surface patient-specific TKA
CPS TKA: Conforming patient-specific TKA
CR: Cruciate-retaining
CT: Computer tomography
FE: Finite element
IE: Internal-external
MPS TKA: Medial pivot patient-specific TKA
MRI: Magnetic resonance imaging
OA: Osteoarthritis
PE: Polyethylene
PID: Proportional-integral-derivative
TKA: Total knee arthroplasty

## References

[CR1] Anderson JG, Wixson RL, Tsai D, Stulberg SD, Chang RW (1996). Functional outcome and patient satisfaction in total knee patients over the age of 75. J Arthroplast.

[CR2] Ardestani MM, Moazen M, Jin Z (2015). Contribution of geometric design parameters to knee implant performance: conflicting impact of conformity on kinematics and contact mechanics. Knee.

[CR3] Asano T, Akagi M, Tanaka K, Tamura J, Nakamura T (2001). In vivo three-dimensional knee kinematics using a biplanar image-matching technique. Clin Orthop Relat Res.

[CR4] Baker PN, van der Meulen JH, Lewsey J, Gregg PJ (2007). The role of pain and function in determining patient satisfaction after total knee replacement. Data from the National Joint Registry for England and Wales. J Bone Joint Surg Br.

[CR5] Blaha JD (2004). The rationale for a total knee implant that confers anteroposterior stability throughout range of motion. J Arthroplast.

[CR6] Blankevoort L, Huiskes R (1996). Validation of a three-dimensional model of the knee. J Biomech.

[CR7] ConforMIS. Inc. http://www.conformis.com. Accessed 30 May 2019.

[CR8] D’Lima DD, Poole C, Chadha H, Hermida JC, Mahar A, Colwell CW (2001). Quadriceps moment arm and quadriceps forces after total knee arthroplasty. Clin Orthop Relat Res.

[CR9] Dennis DA, Mahfouz MR, Komistek RD, Hoff W (2005). In vivo determination of normal and anterior cruciate ligament-deficient knee kinematics. J Biomech.

[CR10] Devers BN, Conditt MA, Jamieson ML, Driscoll MD, Noble PC, Parsley BS (2011). Does greater knee flexion increase patient function and satisfaction after total knee arthroplasty?. J Arthroplast.

[CR11] Godest AC, Beaugonin M, Haug E, Taylor M, Gregson PJ (2002). Simulation of a knee joint replacement during a gait cycle using explicit finite element analysis. J Biomech.

[CR12] Greene KA (2007). Gender-specific design in total knee arthroplasty. J Arthroplast.

[CR13] Grood ES, Suntay WJ (1983). A joint coordinate system for the clinical description of three-dimensional motions: application to the knee. J Biomech Eng.

[CR14] Halloran JP, Clary CW, Maletsky LP, Taylor M, Petrella AJ, Rullkoetter PJ (2010). Verification of predicted knee replacement kinematics during simulated gait in the Kansas knee simulator. J Biomech Eng.

[CR15] Harrysson OL, Hosni YA, Nayfeh JF (2007). Custom-designed orthopedic implants evaluated using finite element analysis of patient-specific computed tomography data: femoral-component case study. BMC Musculoskelet Disord.

[CR16] Haut Donahue TL, Hull M, Rashid MM, Jacobs CR (2003). How the stiffness of meniscal attachments and meniscal material properties affect tibio-femoral contact pressure computed using a validated finite element model of the human knee joint. J Biomech.

[CR17] Hill PF, Vedi V, Williams A, Iwaki H, Pinskerova V, Freeman MA (2000). Tibiofemoral movement 2: the loaded and unloaded living knee studied by MRI. J Bone Joint Surg Br.

[CR18] Ivie CB, Probst PJ, Bal AK, Stannard JT, Crist BD, Sonny Bal B (2014). Improved radiographic outcomes with patient-specific total knee arthroplasty. J Arthroplast.

[CR19] Jaffry Z, Masjedi M, Clarke S, Harris S, Karia M, Andrews B, Cobb J (2014). Unicompartmental knee arthroplasties: robot vs. patient specific instrumentation. Knee.

[CR20] Kang KT, Kim SH, Son J, Lee YH, Chun HJ (2016). Computational model-based probabilistic analysis of in vivo material properties for ligament stiffness using the laxity test and computed tomography. J Mater Sci Mater Med.

[CR21] Kang KT, Kim SH, Son J, Lee YH, Kim S, Chun HJ (2017). Probabilistic evaluation of the material properties of the in vivo subject-specific articular surface using a computational model. J Biomed Mater Res B Appl Biomater.

[CR22] Kang KT, Koh YG, Jung M, Nam JH, Son J, Lee YH, Kim SH (2017). The effects of posterior cruciate ligament deficiency on posterolateral corner structures under gait- and squat-loading conditions: a computational knee model. Bone Joint Res.

[CR23] Kang KT, Koh YG, Son J, Kim SJ, Choi S, Jung M, Kim SH (2017). Finite element analysis of the biomechanical effects of 3 posterolateral corner reconstruction techniques for the knee joint. Arthroscopy.

[CR24] Kang KT, Koh YG, Son J, Kwon OR, Baek C, Jung SH, Park KK (2016). Measuring the effect of femoral malrotation on knee joint biomechanics for total knee arthroplasty using computational simulation. Bone Joint Res.

[CR25] Kang KT, Koh YG, Son J, Kwon OR, Lee JS, Kwon SK (2018). Influence of increased posterior Tibial slope in Total knee arthroplasty on knee joint biomechanics: a computational simulation study. J Arthroplast.

[CR26] Koh YG, Son J, Kwon SK, Kim HJ, Kwon OR, Kang KT (2017). Preservation of kinematics with posterior cruciate-, bicruciate- and patient-specific bicruciate-retaining prostheses in total knee arthroplasty by using computational simulation with normal knee model. Bone Joint Res.

[CR27] Kurtz WB, Slamin JE, Doody SW (2016). Bone preservation in a novel patient specific total knee replacement. Reconstr Rev.

[CR28] Kutzner I, Heinlein B, Graichen F, Bender A, Rohlmann A, Halder A, Bergmann G (2010). Loading of the knee joint during activities of daily living measured in vivo in five subjects. J Biomech.

[CR29] Kwon OR, Kang KT, Son J, Kwon SK, Jo SB, Suh DS, Koh YG (2014). Biomechanical comparison of fixed- and mobile-bearing for unicomparmental knee arthroplasty using finite element analysis. J Orthop Res.

[CR30] Li K, Ackland DC, McClelland JA, Webster KE, Feller JA, de Steiger R, Pandy MG (2013). Trunk muscle action compensates for reduced quadriceps force during walking after total knee arthroplasty. Gait Posture.

[CR31] Losina E, Thornhill TS, Rome BN, Wright J, Katz JN (2012). The dramatic increase in total knee replacement utilization rates in the United States cannot be fully explained by growth in population size and the obesity epidemic. J Bone Joint Surg Am.

[CR32] Mannion AF, Kampfen S, Munzinger U, Kramers-de Quervain I (2009). The role of patient expectations in predicting outcome after total knee arthroplasty. Arthritis Res Ther.

[CR33] Mizner RL, Petterson SC, Stevens JE, Vandenborne K, Snyder-Mackler L (2005). Early quadriceps strength loss after total knee arthroplasty. The contributions of muscle atrophy and failure of voluntary muscle activation. J Bone Joint Surg Am.

[CR34] Noble PC, Gordon MJ, Weiss JM, Reddix RN, Conditt MA, Mathis KB (2005). Does total knee replacement restore normal knee function?. Clin Orthop Relat Res.

[CR35] Parsley BS, Bertolusso R, Harrington M, Brekke A, Noble PC (2010). Influence of gender on age of treatment with TKA and functional outcome. Clin Orthop Relat Res.

[CR36] Patil S, Bunn A, Bugbee WD, Colwell CW, D’Lima DD (2015). Patient-specific implants with custom cutting blocks better approximate natural knee kinematics than standard TKA without custom cutting blocks. Knee.

[CR37] Peña E, Calvo B, Martinez MA, Palanca D, Doblaré M (2006). Why lateral meniscectomy is more dangerous than medial meniscectomy. A finite element study. J Orthop Res.

[CR38] Slamin J, Parsley B (2012). Evolution of customization design for total knee arthroplasty. Curr Rev Musculoskelet Med.

[CR39] Steklov N, Slamin J, Srivastav S, D’Lima D (2010). Unicompartmental knee resurfacing: enlarged tibio-femoral contact area and reduced contact stress using novel patient-derived geometries. Open Biomed Eng J.

[CR40] Stoddard JE, Deehan DJ, Bull AM, McCaskie AW, Amis AA (2013). The kinematics and stability of single-radius versus multi-radius femoral components related to mid-range instability after TKA. J Orthop Res.

[CR41] Takeda Y, Xerogeanes JW, Livesay GA, Fu FH, Woo SL (1994). Biomechanical function of the human anterior cruciate ligament. Arthroscopy.

[CR42] Thompson JA, Hast MW, Granger JF, Piazza SJ, Siston RA (2011). Biomechanical effects of total knee arthroplasty component malrotation: a computational simulation. J Orthop Res.

[CR43] Van Den Heever DJ, Scheffer C, Erasmus P, Dillon E (2011). Contact stresses in a patient-specific unicompartmental knee replacement. Clin Biomech (Bristol, Avon).

[CR44] Varadarajan KM, Zumbrunn T, Rubash HE, Malchau H, Muratoglu OK, Li G (2015). Reverse engineering nature to design biomimetic total knee implants. J Knee Surg.

[CR45] Walker PS, Lowry MT, Kumar A (2014). The effect of geometric variations in posterior-stabilized knee designs on motion characteristics measured in a knee loading machine. Clin Orthop Relat Res.

[CR46] Wang H, Foster J, Franksen N, Estes J, Rolston L (2017). Gait analysis of patients with an off-the-shelf total knee replacement versus customized bi-compartmental knee replacement. Int Orthop.

[CR47] Wünschel M, Lo J, Dilger T, Wulker N, Muller O (2011). Influence of bi- and tri-compartmental knee arthroplasty on the kinematics of the knee joint. BMC Musculoskelet Disord.

[CR48] Yue B, Varadarajan KM, Moynihan AL, Liu F, Rubash HE, Li G (2011). Kinematics of medial osteoarthritic knees before and after posterior cruciate ligament retaining total knee arthroplasty. J Orthop Res.

[CR49] Zeller IM, Sharma A, Kurtz WB, Anderle MR, Komistek RD (2017). Customized versus patient-sized cruciate-retaining Total knee arthroplasty: an in vivo kinematics study using Mobile fluoroscopy. J Arthroplast.

[CR50] Zumbrunn T, Duffy MP, Rubash HE, Malchau H, Muratoglu OK, Varadarajan KM (2018). ACL substitution may improve kinematics of PCL-retaining total knee arthroplasty. Knee Surg Sports Traumatol Arthrosc.

[CR51] Zumbrunn Thomas, Varadarajan Kartik Mangudi, Rubash Harry E., Malchau Henrik, Li Guoan, Muratoglu Orhun K. (2015). Regaining Native Knee Kinematics Following Joint Arthroplasty: A Novel Biomimetic Design with ACL and PCL Preservation. The Journal of Arthroplasty.

